# Protocol for a systematic review of the effects of schools and school-environment interventions on health: evidence mapping and syntheses

**DOI:** 10.1186/1471-2458-11-453

**Published:** 2011-06-09

**Authors:** Chris Bonell, Angela Harden, Helene Wells, Farah Jamal, Adam Fletcher, Mark Petticrew, James Thomas, Margaret Whitehead, Rona Campbell, Simon Murphy, Laurence Moore

**Affiliations:** 1 Department of Social and Environmental Health Research, London School of Hygiene and Tropical Medicine, 15-17 Tavistock Place, London WC1H 9SH, UK; 2 Institute for Health and Human Development, University of East London, Water Lane, London E15 4LZ, UK; 3 Social Science Research Unit, Institute of Education, University of London, 20 Bedford Way, London WC1H 0AL, UK; 4 School of Population, Community and Behavioural Sciences, University of Liverpool, Whelan Building, Quadrangle, Brownlow Hill, Liverpool L69 3GB, UK; 5 Department of Social Medicine, University of Bristol, 39 Whatley Road, Bristol BS8 2PS, UK; 6 Cardiff Institute for Society, Health and Ethics, University of Cardiff, 1-3 Museum Place, Cardiff CF10 3BD, UK

## Abstract

**Background:**

Schools may have important effects on students' and staff's health. Rather than treating schools merely as sites for health education, 'school-environment' interventions treat schools as settings which influence health. Evidence concerning the effects of such interventions has not been recently synthesised.

**Methods/design:**

Systematic review aiming to map and synthesise evidence on what theories and conceptual frameworks are most commonly used to inform school-environment interventions or explain school-level influences on health; what effects school-environment interventions have on health/health inequalities; how feasible and acceptable are school-environment interventions; what effects other school-level factors have on health; and through what processes school-level influences affect health.

We will examine interventions aiming to promote health by modifying schools' physical, social or cultural environment via actions focused on school policies and practices relating to education, pastoral care and other aspects of schools beyond merely providing health education. Participants are staff and students age 4-18 years.

We will review published research unrestricted by language, year or source. Searching will involve electronic databases including Embase, ERIC, PubMed, PsycInfo and Social Science Citation Index using natural-language phrases plus reference/citation checking.

Stage 1 will map studies descriptively by focus and methods. Stage 2 will involve additional inclusion criteria, quality assessment and data extraction undertaken by two reviewers in parallel. Evidence will be synthesised narratively and statistically where appropriate (undertaking subgroup analyses and meta-regression and where no significant heterogeneity of effect sizes is found, pooling these to calculate a final effect size).

**Discussion:**

We anticipate: finding a large number of studies missed by previous reviews; that non-intervention studies of school effects examine a greater breadth of determinants than are addressed by intervention studies; and that intervention effect estimates are greater than for school-based health curriculum interventions without school-environment components.

## Background

UK young people have among the worst health in Europe and there are marked inequalities in health across the social scale, with considerable implications for later health and economic costs[1,2]. Health education programmes are delivered through the school curriculum and aim to improve knowledge, develop skills and modify peer norms, and are now well-established in schools, addressing health behaviours such as smoking, drinking, drug use, sexual behaviour, physical activity and diet, However, numerous systematic reviews suggest such interventions have mixed and frequently disappointing results[3-9].

A complementary approach to curriculum-based health education is to change the school environment to promote health and wellbeing. The physical, social and cultural environment in which staff and students spend a high proportion of every weekday may have profound effects on their emotional and mental health, and opportunities to choose healthy lifestyles. Rather than treating schools merely as sites for health education, school-environment' (SE) interventions aim to modify how the school environment influences health. SE interventions can address health directly, for example: modifying school policies on smoking[10] etc; improving catering[11]; or encouraging staff and students to walk or cycle to school[12]. Other actions aim to address factors such as disengagement and lack of social support that are risk factors for multiple adverse outcomes[13,14]. The latter include: increasing student participation in decision-making; providing staff with training on how to re-engage disaffected students; and encouraging students to take on new responsibilities such as becoming peer mediators[15]. These interventions take a 'socio-ecological' [16] approach to promoting health, whereby health is understood to be influenced not only by individual characteristics and behaviours, but also the wider social, cultural and economic context.

An important influence on the development of SE interventions has been the World Health Organisation's (WHO) framework for 'Health Promoting Schools' (HPS)[17]. This requires that schools simultaneously address their 'ethos' (i.e. school values and priorities), family/community involvement and curriculum. Some HPS have been rigorously evaluated but many have not[18]. Other trials have evaluated interventions which aim to modify the school environment to promote health but which are not explicitly informed by the HPS framework.

Evidence concerning the effects of SE interventions has not been comprehensively synthesised and several reviews that have examined these interventions are now quite old. A decade-old systematic review, focused only on HPS interventions, identifying only 12 studies, four of which were randomized trials. It concluded HPS interventions are promising, especially for promoting healthy eating, reducing bullying and improving mental and social wellbeing [18]. Other systematic reviews have focused on SE interventions that aim to reduce violence and drug use (not explicitly informed by the HPS framework) [19-22]. No evidence syntheses have been done on the effects of SE interventions in important areas such as sexual health, alcohol or smoking.

There has also been no synthesis of evidence on intervention process. Process evaluations examine the planning, delivery and receipt of SE interventions, and are useful for informing decisions about the wider implementation of interventions[23,24]. A further gap concerns synthesis of evidence on the health effects of the normal school environment (i.e. in the absence of intervention). This is important because to date SE intervention studies appear to have addressed only some aspects of the school environment and neglected others, such as school leadership and approaches to learning. Examining the impacts of such factors on health outcomes is now a growing field of public-health research[25] which merits synthesis. Although such studies provide less certain causal inference than experimental studies, those aiming to minimize confounding and other sources of bias could be used to identify promising areas for future intervention studies. A few reviews of such non-evaluation studies have been conducted but these either examine only certain outcomes or are unsystematic. Systematic reviews of school-level influences on drug use[22] and smoking[26] have concluded there is, respectively, emerging and good evidence that factors such as teacher-student relationships and teaching styles may influence health. One non-systematic review of multi-level studies examined a range of health outcomes and, despite missing several important studies, suggested that strong leadership and high expectations appear to influence various health outcomes[27]. Finally, qualitative research has also been used to explore how staff and students perceive their school environment, and the processes they see as influencing health[28]. This evidence would also be useful in informing future SE interventions but remains unsynthesized.

We will work in close collaboration with colleagues in the Universities of Bristol and Cardiff undertaking a Cochrane review updating the decade-old review of interventions following the HPS framework; protocol available on request. While they focus on HPS interventions, we will examine the broader set of SE interventions and the other forms of evidence described above.

Our research questions are as follows:

RQ1: What theories and conceptual frameworks are most commonly used to inform SE interventions or explain school-level influences on health? What testable hypotheses do these suggest?

RQ2: What are the effects of SE interventions (interventions aiming to aiming to promote health by modifying the school physical, social or cultural environment via actions focused on school policies and practices relating to education, pastoral care, sport, extra-curricular activities, catering, travel to and from school and other aspects of school life) evaluated using experimental and quasi-experimental designs compared with standard school practices on health (physical and emotional/mental health and wellbeing; intermediate health measures such as health behaviours, body mass index, teenage pregnancy; and health-promotion outcomes such as health-related knowledge and attitudes) and health inequalities among school staff and students age 4-18 years? What are their direct and indirect costs?

RQ3: How feasible and acceptable are SE interventions? How does context affect this?

RQ4: What are the effects of other school-level factors on health and health inequalities among school staff and students age 4-18 years examined via multi-level and ecological (school) designs?

RQ5: Through what processes might these school-level influences occur?

## Methods/design

The review will follow existing general criteria for the good conduct and reporting of systematic reviews (e.g. the Centre for Reviews and Dissemination guidelines; Quality of Reporting of Meta-analyses guidelines). It will be carried out in two stages: (1) a descriptive map of available research evidence (which will involve exhaustive searching, application of inclusion and exclusion criteria, detailed coding), plus a preliminary synthesis of theories and conceptual frameworks used to inform SE interventions or explain school-level influences on health (on which we will consult with stakeholders to inform priorities for stage 2); and (2) a series of in-depth syntheses in which the available research will be quality assessed, relevant findings extracted, and statistical and narrative/qualitative methods applied to synthesise findings

### Stage 1: identifying and describing studies

In stage 1 we will include reports, without restrictions on language, date or source, that address each of our research questions.

We will exclude the following:

1. General topic - not about health/wellbeing or disease (including studies solely focused on outcomes concerned only with education).

2. Setting/population - not about the students or staff of schools (i.e. serving those age 4-18).

3. Type of report - not reporting primary research, a review of research or a theory

4. Specific focus

4a (for intervention primary studies) - about an intervention that is neither mainly delivered on the school site nor concerned with travel to and from schools (extra-curricular interventions will be included unless excluded based on any of the criteria below); neither about an intervention aiming to promote health/wellbeing or prevent disease nor reporting on the health/wellbeing outcomes of an intervention; about an intervention only involving: health education, information or counselling (regardless of who delivers this); school nursing, clinics or health checks; or health-related goods (medication, contraception, micronutrients etc), but interventions concerning school catering, sport or active transport would be included; about an intervention targeted only to some students on the basis of health-related needs (but interventions targeted on the basis of educational or social but not health needs would be included).

4b (for non-intervention primary studies) - not a study of the effects of the school environment/school-level factors on health/wellbeing.

4c (for reviews and theoretical research) - not a review or theoretical paper with a focus on the school environment, interventions addressing this or school-level effects.

5. Study type

5a (for intervention (primary) studies) - not an empirical outcome evaluation or process evaluation.

5b (for non-intervention (primary) studies) - not empirically examining SE influences on health/wellbeing; if the study is a quantitative study it will be excluded if it is not reporting on school-level variables (but multi-level analyses including school-level analyses would be included), only reporting on school-level measures of student social (e.g. SES) or demographic (e.g. ethnicity) characteristics or students' social networks (but studies examining student-staff relationships would be included), or only reporting on school-level measures of health education (regardless of who delivers this), school-based clinical health services or interventions targeted on the basis of health-related needs.

5c (for reviews or theoretical research) - not a systematic review with a focus on school environment interventions, interventions to address this or school-level effects AND does not propose an abstracted, generalizable way in which features of schools are causally related to student/staff health.

The type of studies sought by this review are not likely to be reliably indexed in databases with controlled vocabularies. Therefore a very sensitive search will be undertaken using multiple natural language phrases (see Additional file 1 for PubMed search strategy). The first "core" search strategy consists of four sets of terms relating to setting, population, intervention/influence and outcomes. A second search uses a broader set of "non-core" terms covering these same areas. Some additional intervention terms will be added to the key terms as a third search. The intention is to sift the first set very carefully while the second and third set will be sifted more quickly. The following databases will be searched in July-August 2010, with no limits on language or date: Australian Educational Index; British Educational Index; CAB Health; The Campbell Library; CINAHL; Cochrane Controlled Trials Database; Embase; ERIC; Health Management Information Consortium; BSS; PubMed; PsycInfo; Social Policy and Practice (includes Child Data & Social Care Online); Social Science Citation Index (Web of Knowledge); Sociological Abstracts; and Dissertation Abstracts/Index to Theses. Econlit and PAIS were also investigated but trial searches produced no new material.

We will also undertake an intensive process of reference-checking of relevant papers, not only those references cited in the papers, but also looking for those papers which cite our target papers (using Citation Indexing in Web of Knowledge) and the *Related Citations *facility in Medline.

Search results will be downloaded into EPPI-Reviewer 4 software for screening. An inclusion criteria worksheet will be prepared, and each reference screened. Three reviewers will undertake these sifts, initially all three sifting the same studies and meeting to compare answers in initial batches of at least three sets of 50 studies to ensure consistency and more batches if required until the disparities are less than 5%, after which sifting will be done individually.

Studies will be descriptively coded based on title and abstract where possible and on full report where necessary. Included studies will be described by applying a standardized classification system for health promotion research[29] supplemented by new codes. For an initial sample, two reviewers will code independently, compare notes and reach consensus drawing on a third reviewer where necessary. Guidance for reviewers will be refined to remove any ambiguities that arise. Subsequent coding will be done by one reviewer. We will thus develop our evidence map.

Alongside this descriptive mapping, we will undertake a preliminary review of literature addressing RQ1. This synthesis will aim to develop hypotheses to be tested in our stage-2 synthesis regarding RQ2-5. Our review of theory will use thematic synthesis methods[30]. At this stage, we will engage with stakeholders via a workshop involving professionals and parent-governors, and a meeting involving young people. Each of these will review our evidence map and theory synthesis and provide comments that we will use these to inform our setting of hypotheses to be examined in stage 2. Additionally, if we identify a body of evidence of a size incommensurate with the planned scale of this evidence synthesis, we will also consult with these groups to determine priorities for stage 2.

### Stage 2: In-depth syntheses addressing each research question

The final scope of the in-depth syntheses will be informed by our descriptive map, theory synthesis and stakeholder consultation. We will restrict in-depth syntheses to the best available evidence. Inclusion criteria relating to methodological quality will be applied to minimize bias. Where relevant these will be applied to each outcome and not merely to overall studies. Draft methodological inclusion criteria for stage 2 are as follows:

RQ1: Not applicable: already synthesised in stage 1.

RQ2: Prospective design with comparison groups; pre-determined outcomes; control for clustering; control of confounding; no over-adjustment for potential mediators; and reporting on attrition, overall and by group (we will include in the review studies with >30% overall attrition, or >10% between-group differences in attrition, but may exclude these from meta-analyses).

RQ3. Process evaluations will not be excluded on the basis of quality but will be quality-assessed and their findings weighted (see below).

RQ4: Control for clustering; control of school-compositional confounders; no over-adjustment for potential mediators; and reporting on attrition (again we may exclude studies with >30% attrition from meta-analyses). If sufficient studies, we will restrict our attention to multi-level, longitudinal studies which can better control for individual-level confounding and for reverse causality.

RQ5: Qualitative studies will also not be excluded on the basis of quality but will be quality-assessed and their findings weighted (see below).

As in stage 1, criteria will be piloted prior to application. To help assure the review's quality at this stage, pairs of reviewers will first work independently and then compare their decisions before reaching consensus for all reports reviewed, involving a third reviewer where necessary.

We will collect detailed data from, and describe, the included studies addressing RQs2-5. For all studies we will extract data on: study research questions/hypotheses; study site and population; sampling; data collection methods; analysis methods; results; and authors' conclusions. Additional data to be extracted for various study types are listed below.

- Quantitative studies addressing RQs 2 and 4: methods of adjustment for clustering; confounders and methods to control these; attrition rates overall and by study arm (RQ2 only); outcome measures; and effect size estimates (overall and by population socio-economic, gender and ethnic sub-group) and measures of confidence/significance.

- Economic studies addressing RQ2: (depending on what studies are found): intervention costs and indirect resource use; basis, assumptions or perspective taken regarding cost estimates; and (if available) economic measures of cost-effectiveness. In addition, we will extract other relevant data on study design and methods as per those listed above for quantitative studies.

- Qualitative studies addressing RQs 3 and 5: the rationale for the sampling method used; the range of stakeholder perspectives explored; and the transparency of reporting methods and data. For process evaluations we will also examine: part of process examined (planning, delivery, receipt); aspect of process examined (feasibility, fidelity/quality, coverage/accessibility, acceptability, appropriateness/fit with measured/perceived need); and aspect of intervention context examined (e.g. socio-demographic, policy, institutional capacity and collaboration, professional capacity). We have previously developed a tool for examining intervention context[31] which will be considered for use in this review, suitably adapted.

The quality of process evaluations and other qualitative research will be assessed according to a set of recently developed criteria used in an HTA-funded review of school-based interventions[32]. Reviewers will assess studies according to: the appropriateness of the sampling strategy to the evaluation aims; the rigour and, where appropriate, flexibility of data collection; the systematic and comprehensive nature of data analysis; whether findings are grounded in/supported by the data; whether the findings are of sufficient depth and breadth; and whether the perspectives of those involving in planning, delivering and receiving the interventions are adequately examined. A final step in the quality assessment of qualitative studies will be to assign studies two types of 'weight of evidence'. Firstly, reviewers will be asked to assign a weight (low, medium or high) to rate the reliability or trustworthiness of the findings (the extent to which the methods employed were rigorous/could minimise bias and error in the findings). Secondly, reviewers will also be asked to assign an additional weight (low, medium, high) to rate the usefulness of the findings for shedding light on factors relating to the research questions. Guidance will be given to reviewers to help them reach an assessment on each criterion and the final weight of evidence. Similarly, assessment and weighting of the methodological quality of any cost, economic evaluations and econometric studies that we find will be informed by application of existing methods and checklists[33,34].

In synthesising the evidence regarding RQs 2 and 4 we will undertake statistical meta-analysis when studies are sufficiently homogenous in terms of interventions (RQ2) and measures (RQs 2 and 4). Statistical heterogeneity of effects will be assessed using Chi-square tests and the magnitude of statistical heterogeneity will be assessed using the I2 statistic. We will undertake subgroup analyses and meta-regression[35] and where no significant heterogeneity of effect sizes is found, these will be pooled to calculate a final effect size. While these analyses may enable us to hypothesise as to possible causes of differences between studies' findings, some heterogeneity is likely to remain, and any statistical analysis will be accompanied by a narrative synthesis.

Where data allow, our meta-analyses will aim to test hypotheses generated from our preliminary synthesis addressing RQ1. The use of a priori hypotheses from RQ1 will: give us an empirical justification for hypothesising that a given concept might impact on study findings; protect us from 'dredging' the data for spurious statistically significant results; and enable us to critique the selection of covariates that are employed in our included studies.

If the number of outcomes for which meta-analyses is possible exceeds the capacity of this project, we will focus on those outcomes prioritised by our stakeholder meeting. Meta-analysis and subgroup analysis will be conducted using EPPI-Reviewer with Stata 11 being used for any meta-regression. As we anticipate that outcomes will be measured using a range of measurement tools, standardisation of results will be required in the form of standardised mean difference. We also anticipate that most of the studies addressing RQ2 will have used cluster randomised controlled trials, and most of those addressing RQ4 will have used multi-level or ecological (school) designs. We will draw on relevant methods[36] to calculate effects sizes from such studies. We will apply an "equity lens" [37] to the to the effectiveness analysis (conducting sub-group analyses employing meta-regression to examine any differences in impact according to socio-economic status, gender or ethnicity) in order to explore the potential impact of school-environment interventions on health inequalities. The precise hypotheses to be tested in these analyses will be determined by our theory synthesis.

Our synthesis of economic evaluations regarding RQ2 will be guided by what evidence we find. Measures of costs and (if available) indirect resource use and cost-effectiveness will be summarised using tables. If measures of resource use are judged sufficiently homogeneous across studies, and applicable or transferable to the UK context, these will be synthesised using statistical meta-analysis[33]. Measures of costs, indirect resource use and cost-effectiveness collected from studies conducted outside the UK and/or in previous years will be adjusted for currency and inflation to the current UK. These data will be used to inform a narrative synthesis of the principal results of economic analyses, a commentary on economic aspects of school-environment interventions, and the applicability of collected economic evidence to the UK.

Findings from qualitative studies addressing RQs 3 and 5 will be synthesised using narrative methods [30,38,39]. Detailed evidence tables will be prepared to describe the methodological quality of each study, details of the intervention or aspect of schools examined, study site/population and findings. Two reviewers will read and re-read data contained within the evidence tables, apply codes and memos to capture the content of the data, and then group and organise codes into higher-order themes. These themes will be used to generate an explanatory framework to address RQs 3 and 5.

Published reports may be incomplete in a wide range of ways. For example: they may not report sufficient detail about their participants for our equity analysis; they may not present information on all the outcomes that were measured (possibly resulting in outcome reporting bias); they may not provide sufficient information about the intervention for accurate characterisation; and they may not report the necessary statistical information for the calculation of effect sizes. In all cases where there is a danger of missing data affecting our analysis, we will contact authors of papers wherever possible to request additional information. Where this process fails to provide the necessary detail (either because we cannot contact the authors, or they are unable to provide the information we need), we will need to use our judgement as to the most appropriate way forward. Statistical information, such as standard deviations and intra-cluster correlation co-efficients can be imputed from similar studies. We will use imputation where necessary - and defensible - and undertake sensitivity analyses to assess the impact of a range of possible values where this is done. In other instances of missing data (such as missing population information) it may not be possible to include a study in a particular analysis if, for example, it is impossible to classify the population using our equity tool.

Finally, we will draw on our five individual syntheses to produce a draft report. We will then organise stakeholder workshops with professionals and parent-governors, and young people to review our key findings and conclusions. Taking on board the views expressed by stakeholders, we will then finalise our technical report and executive summary, and begin disseminating the research via other means.

## Discussion

We anticipate: finding a large number of studies missed by previous reviews; that non-intervention studies of school effects examine a greater breadth of determinants than are addressed by intervention studies; and that intervention effect estimates are greater than for school-based health curriculum interventions without school-environment components.

## Competing interests

The authors declare that they have no competing interests.

## Authors' contributions

CB conceived and designed the study. AH contributed to the design of the study and in particular the methods relating to review and synthesis of qualitative data. HW and FJ contributed to the design of the sifting and data extraction processes. AF and MP contributed to the design of the synthesis of theory and qualitative data. JT contributed to the planned use of EPPI-Reviewer software and to statistical methods. MW contributed to developing the background and planned outputs of the review. RC, SM and LM contributed to how this review inter-relates to a Cochrane review of multi-component "Health Promoting Schools" interventions. All authors read and approved the final manuscript.

## Pre-publication history

The pre-publication history for this paper can be accessed here:

http://www.biomedcentral.com/1471-2458/11/453/prepub

## Supplementary Material

Additional file 1 **PubMed search strategy**. The MeSH and natural-language search terms used in the PubMed electronic bibliographic database. Click here for file
