# Supplementary material for: Protocol for a systematic review of the effects of schools and school-environment interventions on health: evidence mapping and syntheses
Source: BMC Public Health. 2011 Jun 9;11:453. doi: 10.1186/1471-2458-11-453 (PMC3121641; doi:10.1186/1471-2458-11-453)
Supplement: Additional file 1 — PubMed search strategy. The MeSH and natural-language search terms used in the PubMed electronic bibliographic database. [file 1471-2458-11-453-S1.PDF]

Appendix 1: PubMed search strategy

Search terms

Set 1: Setting

School or schools

Set 2: Population

child* or adolescen* or youth or young people or teen* or student* or pupil* or teacher* or teaching staff or school personnel or school staff or parent*

Set 3A: Key Intervention/ School-level effect - key terms

ADJX = within X words of, in both directions. X determined by trial searches.

ethos

school* ADJ5 climate (exc MeSH climate/ or climate change/)

school* ADJ5 environment

school* ADJ5 culture

school* ADJ3 manag*

school* ADJ3 leader*

school* ADJ5 organization or organisation

school* ADJ5 aggregate*

school* ADJ5 governance

education* context*

inter(-)school variation

inter(-)school differen*

inter(-)school inequalit*

school differen* or differen* between school*

school ADJ2 level

school* ADJ3 varia*

school influence*

school* ADJ3 effect or school* ADJ3 effects

restor* justice

<u>Specific intervention types</u>

school* adj3 (breakfast* or lunch* or dinner* or meal*)

breakfast club*

vending

snack* machine* or soft drink* machine*

physical education and training/ MeSH

physical train*

games adj3 school*

sport* adj3 school*

active transport (not cell*)

active commuting to school

walking bus* or walking school bus*

school travel plan*

active commuting to school

walk* ADJ3 school*

cycl* ADJ3 school*

<u>MeSH</u>

Schools/organization and administration

AND Health promotion/

Set 4: Outcome terms

General health/well being terms

Health

Well(-)being or wellbeing

Infection

Disease*

Specific areas

Emotion*

Mental

Psychiatr*

Anxi*

Depress*

Violence

MeSH

Juvenile delinquency/

Violence/*prevention & control

Violen*

Delinquen*

Aggress*

Bully*

Bullies or bullied

Injur*

Accident

Victimi*

<u>Substance abuse</u>

MeSH

Alcohol Drinking/

Marijuana Smoking/

Smoking/

Substance-Related Disorders/*prevention & control

Substance* ADJ2 (use* or abuse* or misuse*)

Smok* or Tobacco or Cigarette*

Drug* ADJ2 (use* or abuse* or misuse*)

Illicit drug* or Illegal drug* or Street drug*

Cannabis or Marijuana

Alcohol

Binge

<u>Obesity issues</u>

Healthy(-)eating

Nutrition

Obesity

Diet

Over(-)weight

Body weight or bodyweight

Body mass or bodymass

Physical exercise

Physical* activi*

Pysical train*

Active Transport

active transport (not cell*)

active commuting to school

walking bus*

school travel plan*

active commuting to school

walk* ADJ3 school*

cycl* ADJ3 school*

Sexual behaviour

MeSH

Acquired Immunodeficiency Syndrome/ epidemiology/*prevention & control

Condoms/utilization

HIV Infections/epidemiology/*prevention & control

Pregnancy in adolescence/

Sexual behavior/

Sexually Transmitted Diseases/epidemiology/*prevention & control

Pregnan*

Sexual

HIV

Chlamydia

Condom [use]

Contracepti*

All excluding MeSh: exp: schools, medical/

or medical school*

NON-CORE TERMS

Set 3b: Intervention/ School-level effect – other non-key terms

General free-text

multi(-)intervention

non-curric*

socio(-)ecological*

ecological or ecology

socio(-)environment

classroom management

value(-)added

engag* or disengag*

student-led

pupil-led

pastoral (not agric* or farm*)

school ADJ3 achievement*

school* ADJ3 attainment*

school* ADJ3 exam*

school* ADJ3 (test or tests or testing or tested)

school* ADJ3 qualif*

school* ADJ3 quality

school* ADJ3 inspect*

school* ADJX influence

school* ADJ5 (policy or policies)

school* ADJ3 rules

school* ADJ5 context*

school* ADJX opport*

school* ADJ3 practices

school* ADJ5 collective

school* ADJ3 communit*

school* ADJ5 structur*

school* ADJ3 relation*

school* ADJ5 communicat*

school* ADJ5 aggregate*

school* ADJ5 security

school* ADJ5 safe*

school* ADJX expectation*

between ADJ2 schools

school* adj2 exclusion*

school inclusion

education* ADJ3 achievement

education* ADJ3 attainment

education* ADJ3 examin*

education* ADJ3 (test* or tests or testing)

education* ADJ3 qualif*

education* ADJ3 quality

education* ADJ3 engag*

education* ADJ3 (policy or policies)

education* ADJ3 opportun*

education* ADJ3 practices

education* ADJ3 culture

education* ADJ3 manag*

education* ADJ3 leader*

education* ADJ3 communicat*

education* ADJ3 safe*

education* ADJ3 expecation*

teaching ADJ3 practices

teaching ADJ3 standard*

teaching ADJ3 style*

teaching ADJ3 method*

teaching ADJ3 differen*

teaching ADJ3 varia*

aggregate* adj1 (data or reports or information)

school* size

school restructur*

comprehensive school reform

vending

School meal*

SET 5 Simple phrases - searched alone

health(-)promoting school*

healthy(-)school or healthy schools

comprehensive school* health program*

co(-)ordinated school* health program*

SET 6 Simple phrases combined with SET 4 outcome terms

school(-)wide

whole(-)school

Search summary

Search One: Set 1 and Set 2 and Set 3a and Set 4 (setting/population and key intervention/effects and outcomes)

Search Two: Set 5 (HPS phrases)

Search Three: Set 6 and Set 4 (whole school phrases and outcomes)

Search Four: Set 1 and Set 2 and Set 3b and Set 4 (setting/population and key intervention/effects and outcomes)
